# Efficient cell-free expression with the endogenous *E. Coli *RNA polymerase and sigma factor 70

**DOI:** 10.1186/1754-1611-4-8

**Published:** 2010-06-24

**Authors:** Jonghyeon Shin, Vincent Noireaux

**Affiliations:** 1 University of Minnesota, 116 Church Street S.E., Minneapolis, MN 55455, USA

## Abstract

**Background:**

*Escherichia coli *cell-free expression systems use bacteriophage RNA polymerases, such as T7, to synthesize large amounts of recombinant proteins. These systems are used for many applications in biotechnology, such as proteomics. Recently, informational processes have been reconstituted *in vitro *with cell-free systems. These synthetic approaches, however, have been seriously limited by a lack of transcription modularity. The current available cell-free systems have been optimized to work with bacteriophage RNA polymerases, which put significant restrictions to engineer processes related to biological information. The development of efficient cell-free systems with broader transcription capabilities is required to study complex informational processes *in vitro*.

**Results:**

In this work, an efficient cell-free expression system that uses the endogenous *E. coli *RNA polymerase only and sigma factor 70 for transcription was prepared. Approximately 0.75 mg/ml of *Firefly luciferase *and enhanced green fluorescent protein were produced in batch mode. A plasmid was optimized with different regulatory parts to increase the expression. In addition, a new eGFP was engineered that is more translatable in cell-free systems than the original eGFP. The protein production was characterized with three different adenosine triphosphate (ATP) regeneration systems: creatine phosphate (CP), phosphoenolpyruvate (PEP), and 3-phosphoglyceric acid (3-PGA). The maximum protein production was obtained with 3-PGA. Preparation of the crude extract was streamlined to a simple routine procedure that takes 12 hours including cell culture.

**Conclusions:**

Although it uses the endogenous *E. coli *transcription machinery, this cell-free system can produce active proteins in quantities comparable to bacteriophage systems. The *E. coli *transcription provides much more possibilities to engineer informational processes *in vitro*. Many *E. coli *promoters/operators specific to sigma factor 70 are available that form a broad library of regulatory parts. In this work, cell-free expression is developed as a toolbox to design and to study synthetic gene circuits *in vitro*.

## Background

Cell-free systems are used to synthesize large amounts of recombinant proteins in a few hours. Cell-free expression, which has many advantages in comparison to cell-based expression, is employed in an increasing number of biotechnology and proteomic applications [1]. Many efforts are made to increase the protein productivity and the functionality of cell-free systems. The energy regeneration is frequently optimized [2-6], new *E. coli *strains are engineered to stabilize some amino acids or to express proteins with PCR products [7,8] and preparation of the crude extract is simplified [9]. Current extracts prepared from *E. coli *cells can deliver between half and one milligram per milliliter of reporter protein after a few hours of incubation in a test tube. To achieve high protein yield, these extracts use bacteriophage RNA polymerases for transcription, such as T7 and T3, with their specific promoters, because they are the most efficient polymerases. Although well established, however, cell-free expression is narrowed to bacteriophage transcription and is not necessarily adapted to all types of applications. Bacteriophage transcription cannot replace endogenous RNA polymerases for the synthesis of particular genes [10]. In cell-free systems, bacteriophage transcription has to be modified to improve expression of specific genes [11]. Reconstitution of cell-free informational processes, and on a broader range the field of *in vitro *synthetic biology, is limited to bacteriophage RNA polymerases [12-17]. Only a few synthetic bacteriophage promoters regulated by operators have been characterized, such as the T7/lacO system. The reconstitution of informational processes *in vitro *would greatly benefit from having efficient cell-free systems that offer a greater modularity at the level of transcription. Promoter/operators from *E. coli *provide much more modularity for transcription [18]. *E. coli *cell-free extracts working with the endogenous core RNA polymerase and the housekeeping sigma factor 70 were originally prepared to study gene expression mechanisms [19,20]. However, the endogenous transcription of *E. coli *extract, which is slower and less efficient than bacteriophage transcription, is not believed to allow efficient *in vitro *synthesis of proteins. For this reason, bacteriophage transcription has been largely favored at the expense of endogenous transcription systems.

In this work, the preparation of an efficient cell-free expression system that uses the endogenous *E. coli *RNA polymerase only with sigma factor 70 for transcription is described. Using recent improvements in cell-free preparation and a plasmid with optimized regulatory parts, 0.75 mg/ml (0.6 mg/ml active) of *Firefly luciferase *(Luc) and 0.75 mg/ml (0.65 mg/ml active) of enhanced green fluorescent protein (eGFP) were produced. The best protein production was obtained with the 3-PGA energy regenerating system. The expression was also characterized with buffers containing either CP or PEP for the energy regeneration. The preparation of the crude extract, which takes only half a day without freezing the cells, was reduced to a routine procedure faster than the preparation of bacteriophage cell-free systems. A new eGFP cDNA was engineered that allow much higher production of the reporter in cell-free system when it is transcribed from an *E. coli *promoter. The expression and the protein production were characterized with this new enhanced green fluorescent protein and with Luc. This cell-free system can deliver large amounts of proteins without addition of exogenous RNA polymerase.

## Methods

### Extract preparation

The crude extract was prepared with *E. coli *BL21 Rosetta2 cells (Novagen) grown at 37°C in 2xYT medium up to OD600 = 1.5, according to Kigawa et al [21] and Liu et al [9]. All the following steps were performed either on ice or at 4°C except for the pre-incubation at 37°C. The cells were washed twice and resuspended in S30 buffer A (50 mM Tris, 60 mM potassium glutamate, 14 mM magnesium glutamate, pH 7.7, 2 mM DTT). The cells were broken with a bead-beater (Biospecs Products Inc, mini bead-beater-1) using 0.1 mm glass beads. The extract was clarified by centrifugation at 30000 g for 25 minutes. The clear supernatant was pre-incubated 80 minutes at 37°C followed by a centrifugation at 30000 g for 10 minutes. The clear supernatant was dialyzed against S30 buffer B (5 mM Tris, 60 mM potassium glutamate, 14 mM magnesium glutamate, pH 8.2, 1 mM DTT) for 3 hours with a Slide-A-Lyzer cassette (MWCO 10 kDa, Pierce Biotechnology). The crude extract was stored at -80°C after dialysis. A typical concentration of 27-30 mg/ml of proteins in the crude extract was measured by Bradford assay. The crude extract is stable at least 1 year at -80°C.

### Cell-free reaction

The cell-free reactions were composed of 33% crude extract (9-9.5 mg/ml of proteins), the other 66% contained the reaction buffer and the plasmid. The reaction buffers were prepared with slight modifications according to Sitaraman and co-workers for 3-PGA [4], Kim and Swartz for PEP [3] and Kim and co-workers for CP [22]. 3-PGA and PEP are natural *E. coli *substrates, therefore no enzyme was added to the reaction. Creatine phosphate is not a natural substrate for *E. coli*. Creatine kinase was added according to Kim and co-workers [22].

#### (i) 3-PGA buffer

50 mM Hepes pH 8, 1.5 mM ATP and GTP each, 0.9 mM CTP and UTP each, 1 mM spermidine, 0.75 mM cAMP, 0.33 mM NAD, 0.26 mM coenzymeA, 30 mM 3-PGA, 0.068 mM folinic acid, 0.2 mg/ml tRNA, 1 mM IPTG.

#### (ii) PEP buffer

50 mM Hepes pH 8, 1.5 mM ATP and GTP each, 0.9 mM CTP and UTP each, 1 mM putrescine, 0.75 mM cAMP, 0.33 mM NAD, 0.26 mM coenzymeA, 30 mM PEP, 0.068 mM folinic acid, 0.2 mg/ml tRNA, 1 mM IPTG.

#### (iii) CP buffer

50 mM Hepes pH 8, 1.5 mM ATP and GTP each, 0.9 mM CTP and UTP each, 0.5 mM spermidine, 0.75 mM cAMP, 0.33 mM NAD, 0.26 mM coenzymeA, 67 mM CP, 0.0032 mg/ml creatine kinase, 0.068 mM folinic acid, 0.17 mg/ml tRNA, 1 mM IPTG.

The concentrations of amino acids (0.5-1.5 mM for eGFP and 1.5 mM for Luc, for each amino acid), PEG 8000 (0.5-2%), additional magnesium glutamate (0-10 mM) and additional potassium glutamate (0-120 mM) were adjusted depending on the reporter and the buffer used. The optimum plasmid concentration is discussed in the results and discussion section. The volume of each reaction was 10 μl. The reactions were incubated at 22°C for Luc and 29°C for eGFP with no shaking. The reagents were purchased from Sigma, USB Corporation (GTP, CTP, UTP) and Roche (tRNA, amino acids). The concentrations of magnesium and potassium shown in the graphs are additional to the magnesium and potassium provided by the crude extract. The crude extract brings an additional 4.5 mM of magnesium glutamate and 20 mM of potassium glutamate.

### Plasmids

All the plasmids used in this study originate from the plasmid pBEST-Luc (Promega). The list and sequences of the different regulatory parts are reported in the additional file 1 (List on page 1). Luc refers to *Firefly luciferase *[GenBank: CAA59281.1], eGFP to the enhanced green fluorescent protein [GenBank: CAD97424.1], eGFP-Del6 to the enhanced green fluorescent protein truncated and modified in N-terminal, eGFP-Del6-229 to the enhanced green fluorescent protein truncated and modified in N- and C-terminal, PtacI to the consensus *E. coli *sigma factor 70 promoter [23], OR2-OR1-Pr to the lambda repressor Cro promoter [GenBank: J02459.1], UTR1 to the untranslated region containing the T7 *g10 *leader sequence for highly efficient translation initiation [24] [GenBank: M35614.1] and T500 to the transcription terminator [25]. The plasmids were prepared according to the standard molecular cloning procedures. Picogreen (Invitrogen) was used to measure plasmid concentrations. The *E. coli *strain KL740 was purchased from the Coli Genetic Stock Center (Yale) [CGSC#: 4382].

### Measurements

A Wallac Victor III platereader (PerkinElmer) was used to measure eGFP, eGFP-Del6 and eGFP-Del6-229 expression (kinetics and end-point measurements). Pure recombinant eGFP (Clontech) was used for calibration. Luc expression was measured with a custom-built luminometer described previously [12]. Pure Luc and Luc assay kit (Promega) were used for calibration and measurements. Polyacrylamide gel electrophoresis was carried out according to the standard procedures and labeled with SimplyBlue safestain (Invitrogen).

## Results and discussion

### Material and cell-free preparation

The crude extract was prepared according to Kigawa et al [21] and Liu et al [9] with some modifications. The usual buffers S30 A and S30 B used for the preparation of cell-free systems were composed of magnesium glutamate and potassium glutamate instead of the acetate form of the ions [26]. DTT only was used for the washing and the resuspension steps (S30 buffer A) at a concentration of 2 mM instead of the usual combination of 2-mercaptoethanol and DTT. The optimum pH for the S30 buffer A and the S30 buffer B was 7.7 and 8.2 respectively. The cells were broken with a mini bead-beater. This lysis method is more reproducible than the other methods [21] and this technique is also more affordable. Typically, 4.5 g of cells were resuspended in 4.05 ml of S30 buffer A before adding 23 g of dry beads. The mixture was homogenized before bead beating twice at 4600 rpm for 30 seconds with a pause of 30 seconds in between. The mixture was centrifuged at 30000 g for 25 minutes followed by a pre-incubation of the clear supernatant at 37°C for 80 minutes. The extract was clarified again at 30000 g for 10 minutes before 3 hours of dialysis. The entire preparation, from cell culture to crude extract storage, takes 12 hours and does not present any particular constraints compared to other procedures. The cells do not need to be frozen at any time during the process. The crude extract can be thawed once without any loss of activity. Two liters of cell culture give approximately 6 ml of crude extract with a final protein concentration of 27-30 mg/ml.

### Optimization of the reaction

**Figure 1:**
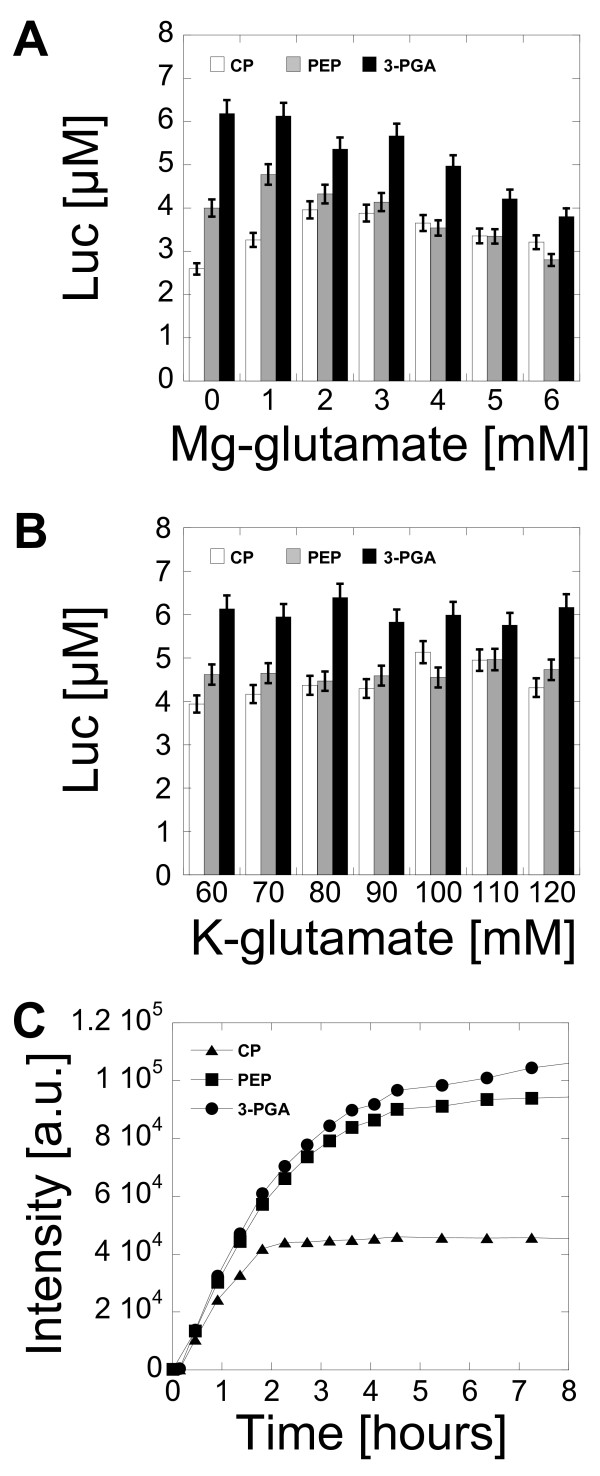
**Protein synthesis as a function of magnesium glutamate and potassium glutamate in three different buffers CP, PEP and 3-PGA**. (A) Luc synthesized as a function of magnesium glutamate with 5 nM plasmid pBEST-UTR1-Luc (CP, PEP and 3-PGA: 90 mM potassium glutamate, 1.5 mM of each amino acid, 2% PEG 8000). (B) Luc synthesized as a function of potassium glutamate with 5 nM plasmid pBEST-UTR1-Luc (CP: 2 mM magnesium glutamate, 1.5 mM of each amino acid, 2% PEG 8000; PEP: 1 mM magnesium glutamate, 1.5 mM of each amino acid, 2% PEG 8000; 3-PGA: 0 mM magnesium glutamate, 1.5 mM of each amino acid, 2% PEG 8000). (C) Kinetics of eGFP expression in the best conditions for the three different buffers with 5 nM plasmid pBEST-UTR1-eGFP (CP: 10 mM magnesium glutamate, 50 mM potassium glutamate, 1 mM of each amino acid, 2% PEG 8000; PEP: 9 mM magnesium glutamate, 30 mM potassium glutamate, 1 mM of each amino acid, 2% PEG 8000; 3-PGA: 6 mM magnesium glutamate, 40 mM potassium glutamate, 1 mM of each amino acid, 2% PEG 8000). Concentrations of magnesium glutamate and potassium glutamate reported here are the concentrations added to the cell-free reaction. The crude extract, dialyzed against the S30 buffer B, brings an additional 4.5 mM magnesium glutamate and 20 mM potassium glutamate to the cell-free reaction.

The cell-free reaction contained all the usual components of bacteriophage systems with minor modifications. Almost all the components in the reaction were optimized. The results for DTT, tRNA, and amino acids are reported in the additional file 1 (Figures S1, S2 and S3). The most important parameters for the optimization of the cell-free reactions were the magnesium concentration, the potassium concentration and the plasmid construction. The magnesium and the potassium concentrations were adjusted with the plasmids pBEST-UTR1-Luc and pBEST-UTR1-eGFP. The reporter genes were transcribed with a PtacI promoter [23]. PtacI, a consensus *E. coli *sigma factor 70 promoter, is one of the strongest *E. coli *promoters. The untranslated region of the plasmid pBEST-Luc was replaced by the untranslated region called UTR1 in this work. The UTR1 sequence includes the T7 gene *10 *leader RNA, which contains a strong ribosome binding site [24]. The lac operator overlapping the -10 part of the promoter was conserved. With this modification, the expression was increased by a factor of five to ten on average in any of the buffers. The protein production was characterized with the three most common ATP regeneration systems used in cell-free studies: creatine phosphate (CP), phosphoenolpyruvate (PEP) and 3-phosphoglyceric acid (3-PGA). In each of these buffers, we found that the concentration of magnesium had to be precisely adjusted in order to maximize protein production (Figure 1A). This result was similar to the results obtained with cell-free systems using bacteriophage RNA polymerases. The best expression was obtained with 3-PGA, followed by PEP and CP. In the three buffers, the protein production was relatively independent of the potassium concentration over a wide range of concentrations (Figure 1B). The best protein production was also obtained with 3-PGA, followed by PEP and CP. For eGFP, the best expression was obtained with the 3-PGA buffer although the results obtained with the PEP buffer were close to 3-PGA (Figure 1C). All the following experiments were carried out with the 3-PGA buffer, which gave a greater protein production than PEP and CP buffers. In the 3-PGA buffer, the protein production was independent of the nucleotide concentrations over a wide range of concentrations (additional file 1, Figure S4.

**Figure 2:**
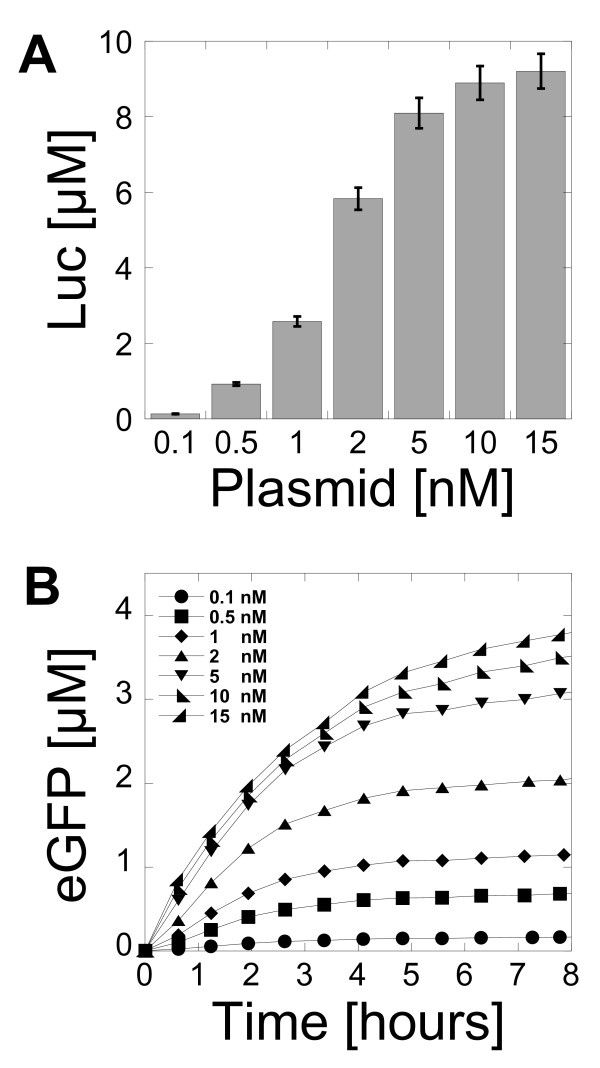
**Protein synthesis as a function of plasmid concentration**. (A) Luc synthesized as a function of plasmid pBEST-UTR1-Luc concentration in 3-PGA buffer (conditions: 0 mM magnesium glutamate, 80 mM potassium glutamate, 1.5 mM of each amino acids, 0.5% PEG 8000). (B) Kinetics of eGFP expression as a function of plasmid pBEST-UTR1-eGFP concentration in 3-PGA buffer (conditions: 4 mM magnesium glutamate, 60 mM potassium glutamate, 1 mM of each amino acids, 2.5% PEG 8000). Concentrations of magnesium glutamate and potassium glutamate reported here are the concentrations added to the cell-free reaction. The crude extract, dialyzed against the S30 buffer B, brings an additional 4.5 mM magnesium glutamate and 20 mM potassium glutamate to the cell-free reaction.

The plasmid concentration is another important parameter in a cell-free reaction. Because the cell-free system described in this study works with the endogenous RNA polymerase and sigma factor 70, it was interesting to determine the quantity of template required to get maximum protein production and to compare it to the amount of plasmid required in bacteriophage-based transcription systems. The best protein production was obtained with a plasmid concentration between 5 nM and 15 nM for Luc (Figure 2A) and eGFP (Figure 2B). Surprisingly, the amount of plasmid required for maximum protein production was comparable to cell-free systems using bacteriophage RNA polymerase for transcription [2-6]. The protein production was linear with respect to the plasmid concentration below 2 nM. At a plasmid concentration of 5 nM, the protein production was 90% of the maximum for Luc and 80% of the maximum for eGFP. In our best extract preparation, a maximum Luc production of 10 μM could be measured, which corresponds to 0.6 mg/ml of active proteins. This protein yield, comparable to bacteriophage cell-free systems, was unexpected because the endogenous *E. coli *transcription machinery is much less efficient. However, the maximum production of eGFP was much smaller than Luc, with only 0.1 mg/ml produced (4 μM). Addition of pure *E. coli *RNA polymerase saturated with sigma factor 70 did not increase the protein production (additional file 1, Figure S5). This led us to the conclusion that expression could be optimized with different DNA regulatory parts and that eGFP was poorly translated. Furthermore, the amplification of plasmids with *E. coli *promoters can be problematic because of potential over-expression of the recombinant proteins. The plasmids pBEST-UTR1-Luc and pBEST-UTR1-eGFP were leaky during plasmid amplification despite the lac operator.

### Plasmid optimization

The maximum production of Luc was obtained with the plasmid pBEST-UTR1-Luc, which contains the PtacI consensus promoter and UTR1 (Figure 2A). No other modifications increased Luc production. A maximum of 0.6 mg/ml (10 μM) active Luc was measured by luminescence. eGFP production with the plasmid pBEST-UTR1-eGFP was significantly lower with only a few micromolars of eGFP produced (Figure 2B). This was far below the expected amount compared to Luc with the same plasmid. We found that translation initiation of eGFP did not work efficiently in our extract. To resolve this problem, the N-terminal sequence of eGFP was truncated according to the work of Li et al [27] and modified by silent mutations to remove ribosome binding site-like sequences. The new protein was named eGFP-Del6. Compared to pBEST-UTR1-eGFP, the fluorescence intensity at the end of the reaction was approximately 3 times greater with the plasmid pBEST-UTR1-eGFP-Del6 (Table 1). This increase, which is a real increase of protein production as opposed to an increase of the reporter fluorescence, was confirmed later by polyacrylamide gel electrophoresis (Figure 4B). The C-terminal sequence of eGFP was also modified [27]. A 20% increase in protein production was measured with this new reporter, named eGFP-Del6-229 (Table 1).

**Table 1:** eGFP and eGFP variants synthesis as a function of DNA regulatory parts

Plasmid pBEST-	Plasmid concentration [nM]	Protein production [μM] (mg/ml)	
eGFP	1	0.02	(0.00067)
UTR1-eGFP	1	1.05	(0.0284)
UTR1-eGFP-T500	1	1.10	(0.0298)
UTR1-eGFP-Del6	1	3.16	(0.0836)
UTR1-eGFP-Del6-229	1	3.72	(0.0944)
UTR1-eGFP-Del6-229-T500	1	4.65	(0.118)
OR2-OR1-Pr-UTR1-eGFP	1	1.40	(0.0375)
OR2-OR1-Pr-UTR1-eGFP-Del6	1	3.30	(0.0874)
OR2-OR1-Pr-UTR1-eGFP-Del6-229	1	4.24	(0.108)
OR2-OR1-Pr-UTR1-eGFP-Del6-229-T500	1	5.07	(0.129)
OR2-OR1-Pr-UTR1-eGFP-Del6-229-T500	2	10.4	(0.264)
OR2-OR1-Pr-UTR1-eGFP-Del6-229-T500	5	20.2	(0.513)
OR2-OR1-Pr-UTR1-eGFP-Del6-229-T500	10	25.2	(0.639)
OR2-OR1-Pr-UTR1-eGFP-Del6-229-T500	15	24.2	(0.615)
OR2-OR1-Pr-UTR1-eGFP-Del6-229-T500	20	23.4	(0.593)

At 1 nM, protein expression with pBEST-OR2-OR1-Pr-UTR1-eGFP-Del6-229-T500 is about 250 times greater than expression with pBEST-eGFP (conditions: 5 mM magnesium glutamate, 50 mM potassium glutamate, 1.5 mM of each amino acid, 2% PEG 8000). Concentrations of magnesium glutamate and potassium glutamate reported here are the concentrations added to the cell-free reaction. The crude extract, dialyzed against the S30 buffer B, brings an additional 4.5 mM magnesium glutamate and 20 mM potassium glutamate to the cell-free reaction.

Cell-free expression with *E. coli *sigma factor 70 promoters can pose significant problems of toxicity due to a potential over-expression of the recombinant protein during plasmid amplification. Although showing some signs of toxicity, the plasmids pBEST-UTR1-Luc and pBEST-UTR1-eGFP-Del6-229 were stable during amplification. A more general solution to this problem was found by switching to the strong Lambda phage promoter Pr flanked by the two operators OR2 and OR1. The *E. coli *strain KL740, which over-expresses the temperature sensitive Lambda repressor Cl857, was used to efficiently repress and stabilize the plasmids during cell growth at temperatures below 30°C. Potential problems due to increased background expression during plasmid amplification could also be solved by changing the origin of replication. The plasmid pBEST-Luc has a ColE1 origin of replication (few hundreds copies per cell), which can be replaced by a P15A (10-12 copies per cell) or a PSC101 (2-5 copies per cell) origin of replication. Other tightly regulated transcription systems and strains could be also used [28].

**Figure 3:**
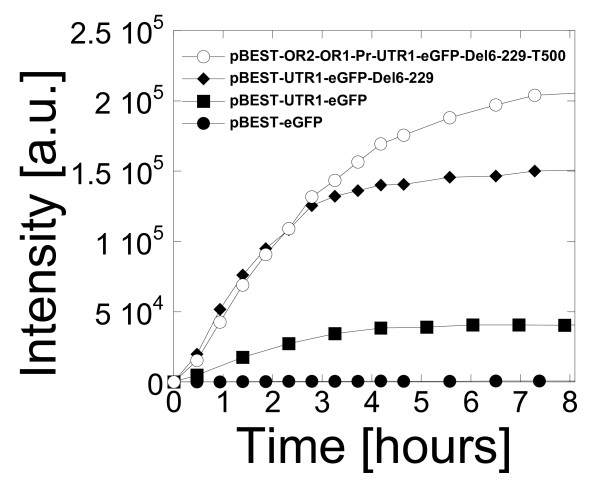
**Cell-free protein synthesis of eGFP variants as a function of DNA regulatory parts**. Expression kinetics of four eGFP variants, 1 nM plasmid final concentration (3-PGA buffer, conditions: 5 mM magnesium glutamate, 50 mM potassium glutamate, 1.5 mM of each amino acid, 2% PEG 8000). Concentrations of magnesium glutamate and potassium glutamate reported here are the concentrations added to the cell-free reaction. The crude extract, dialyzed against the S30 buffer B, brings an additional 4.5 mM magnesium glutamate and 20 mM potassium glutamate to the cell-free reaction.

In the case of eGFP-Del6-229, expression with the OR2-OR1-Pr promoter was slightly greater than with the PtacI promoter (Table 1). A 20% increase in eGFP-Del6-229 production was observed with the addition of the strong transcription terminator T500 [25]. For eGFP-Del6-229, the best expression was obtained with the plasmid pBEST-OR2-OR1-Pr-UTR1-eGFP-Del6-229-T500 at a concentration of 10 nM (Table 1). In the best conditions, a concentration of 0.65 mg/ml of active eGFP-Del6-229 was measured. The protein was synthesized at the same rate whether transcribed from a PtacI or a OR2-OR1-Pr promoter (Figure 3). However, the expression lasted a longer time with the lambda promoter. The production of eGFP with the original plasmid pBEST-eGFP was more than 100 times smaller than with the plasmid pBEST-OR2-OR1-Pr-UTR1-eGFP-Del6-229-T500.

**Figure 4:**
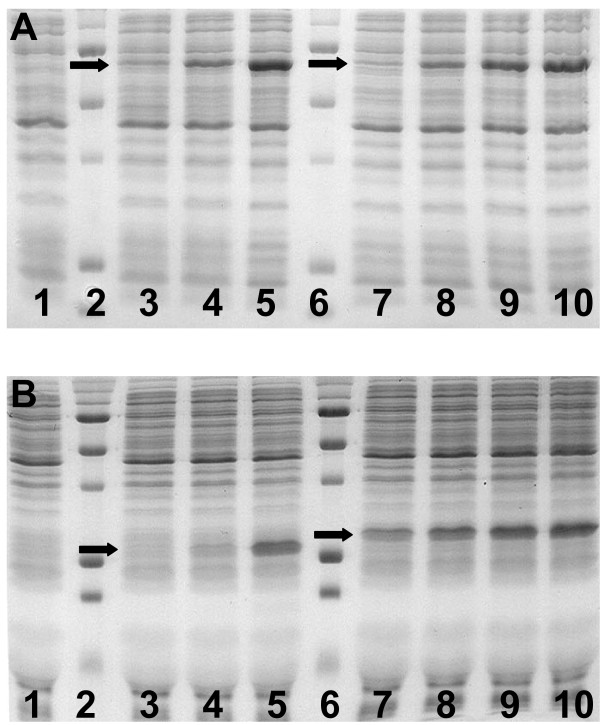
**Analysis of protein production by polyacrylamide gel electrophoresis**. For both gel, lane 1 is a negative control (no plasmid added in the reaction), lane 2 and 6 is a protein ladder (5 main bands from top to bottom 75, 50, 37, 25 and 20 kDa). Synthesized proteins are indicated by arrows. (A) Luc synthesized with 10 nM pBEST-Luc plasmid (lane 3), 1.5 nM pBEST-UTR1-Luc plasmid (lane 4), and 15 nM pBEST-UTR1-Luc plasmid (lane 4) (3-PGA buffer, conditions: 1 mM magnesium glutamate, 60 mM potassium glutamate, 1.5 mM of each amino acid, 0.5% PEG 8000). Pure Luc added to the cell-free reaction with no plasmid, 2 μM (lane 7), 6 μM (lane 8), 10 μM (lane 9) and 14 μM (lane 10). (B) eGFP synthesized with 15 nM pBEST-eGFP plasmid (lane 3), 1 nM pBEST-OR2-OR1-Pr-UTR1-eGFP-Del6-229-T500 plasmid (lane 4) and 10 nM pBEST-OR2-OR1-Pr-UTR1-eGFP-Del6-229-T500 plasmid (lane 5), (3-PGA buffer, conditions: 3 mM magnesium glutamate, 30 mM potassium glutamate, 1.5 mM of each amino acid, 2% PEG 8000). Pure eGFP added to the cell-free reaction with no plasmid, 10 μM (lane 7), 15 μM (lane 8), 20 μM (lane 9) and 25 μM (lane 10). Concentrations of magnesium glutamate and potassium glutamate reported here are the concentrations added to the cell-free reaction. The crude extract, dialyzed against the S30 buffer B, brings an additional 4.5 mM magnesium glutamate and 20 mM potassium glutamate to the cell-free reaction.

The high protein production for both Luc and eGFP-Del6-229 was confirmed by polyacrylamide gel electrophoresis (Figure 4A and 4B). SDS PAGE analysis showed that eGFP-Del6-229 production was greater than eGFP production. This was essentially due to the modification of the N-terminal of the protein (Table 1). An amount of 0.75 mg/ml for both Luc and eGFP-Del6-229 could be estimated on gel whereas the amount of active reporter proteins measured by luminescence was 0.60 mg/ml (Luc) and by fluorescence 0.65 mg/ml (eGFP-Del6-229). The plasmid maps for pBEST-UTR1-Luc and pBEST-OR2-OR1-Pr-UTR1-eGFP-Del6-229-T500 can be found in the additional file 1 (Figure S6).

The protein productions measured in this work were comparable to the protein productions obtained with cell-free systems using bacteriophage RNA polymerases. Expression of GFP with a T7 transcription, characterized and quantified in two cell-free systems by Iskakova and co-workers [11], was comprised between 0.3 and 0.7 mg/ml. A maximum of 0.15 mg/ml of active Luc was measured in two different cell-free systems [12,14]. Furthermore, the protein production obtained with commercial cell-free systems based on bacteriophage transcription is comprised between 0.5 and 1 mg/ml.

With the CP buffer, the maximum Luc and eGFP-Del6-229 production was only 60% of the amount obtained with 3-PGA. With the PEP buffer, the maximum Luc and eGFP-Del6-229 production was 60% and 100% respectively of the amount obtained with 3-PGA. It is important to note that Luc production was neither greater nor smaller with the different regulatory parts tested with eGFP. The production of Luc with the plasmids pBEST-UTR1-Luc and pBEST-OR2-OR1-Pr-UTR1-Luc-T500 were identical. The plasmid pBEST-OR2-OR1-Pr-UTR1-Luc-T500 was easier to amplify in the *E. coli *strain KL740.

### Expression of reporters as a function of temperature

**Figure 5:**
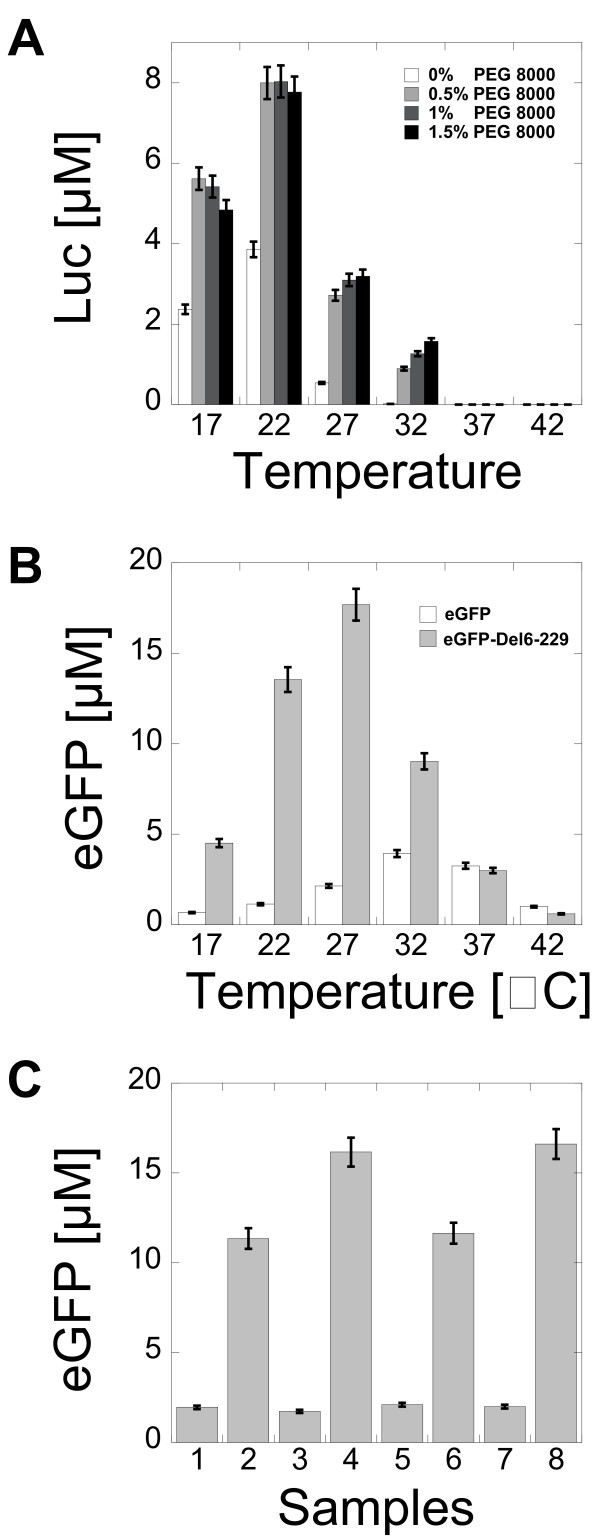
**Luc, eGFP and eGFP-Del6-229 production as a function of temperature**. (A) End-point measurement of Luc production as a function of PEG 8000 concentration and as a function of the reaction incubation temperature (3-PGA buffer, conditions: 0 mM magnesium glutamate, 80 mM potassium glutamate, 1.5 mM of each amino acid). (B) End-point measurement of eGFP and eGFP-Del6-229 production as a function of the reaction incubation temperature (3-PGA buffer, conditions: 4 mM magnesium glutamate, 60 mM potassium glutamate, 1.5 mM of each amino acid, 2.5% PEG 8000). (C) Temperature stability of eGFP and eGFP-Del6-229 protein expressed with 2 nM plasmids (pBEST-UTR1-eGFP-T500 and pBEST-UTR1-eGFP-Del6-229-T500). 1) 8 hours expression of eGFP at 32°C, 2) 8 hours expression of eGFP-Del6-229 at 32°C, 3) 8 hours expression of eGFP at 29°C, 4) 8 hours expression of eGFP-Del6-229 at 29°C, 5) 8 hours expression of eGFP at 32°C followed by 1 hour of incubation at 37°C, 6) 8 hours expression of eGFP-Del6-229 at 32°C followed by 1 hour of incubation at 37°C, 7) 8 hours expression of eGFP at 29°C followed by 1 hour of incubation at 37°C, 8) 8 hours expression of eGFP-Del6-229 at 29°C followed by 1 hour of incubation at 37°C. Concentrations of magnesium glutamate and potassium glutamate reported here are the concentrations added to the cell-free reaction. The crude extract, dialyzed against the S30 buffer B, brings an additional 4.5 mM magnesium glutamate and 20 mM potassium glutamate to the cell-free reaction.

The protein production in cell-free reactions depends on the temperature of incubation. For Luc, the best protein production was obtained in the temperature range 20-25°C (Figure 5A). This result was in agreement with previous studies on the reporter's activity that showed a net decrease of luminescence above room temperature [29-31]. In the case of eGFP, the best protein production was obtained at 32°C (Figure 5B). At this temperature, the expression of eGFP-Del6-229 was twice greater than eGFP. The best production of eGFP-Del6-229 was obtained at 29°C. At 37°C and 42°C, expression of eGFP and eGFP-Del6-229 were comparable. Additional tests were performed to determine if the temperature range 29-32°C was optimum for gene expression or optimum for thermo-stability of the proteins. Cell-free reactions were carried out at 29°C and 32°C for both eGFP and eGFP-Del6-229 during 8 hours followed by 1 hour of incubation at 37°C (Figure 5C). No change in fluorescence was observed. This result confirmed that the production of fluorescent proteins was maximum in the temperature range 29-32°C. Similar results have been obtained for the wild type GFP [11]. In the best conditions, eGFP production never reached more than 25% of the maximum obtained for eGFP-Del6-229. The expression of eGFP and eGFP-Del6-229 was also tested in *E. coli *using the same plasmid (pBEST-UTR1 with a PtacI promoter). The expression of eGFP-Del6-229 was slightly better than eGFP (additional file 1, Figure S7).

## Conclusions

In this work, an efficient cell-free expression system driven only by the endogenous *E. coli *RNA polymerase was prepared. Surprisingly, the protein production with this extract is comparable to bacteriophage systems. This new system is as workable as bacteriophage systems and does present any particular constraints. In order to obtain efficient protein production, the reaction conditions and its components were optimized as well as the regulatory parts of the plasmid carrying the recombinant gene. Our work, however, does not exclude further improvements. Other promoters, untranslated regions and terminators could be tested to increase the protein production. We used the three most common ATP regeneration systems, 3-PGA, PEP and CP. Cell-free protein synthesis with this system, however, could also benefit from recent results at the energy regeneration level [6]. In the course of this study, we found that eGFP was not translated efficiently when it was expressed *in vitro *from an *E. coli *promoter. The gene encoding the reporter was modified to get highly translatable reporter messenger RNAs. This cell-free system opens new possibilities to synthesize proteins and it broadens the range of potential applications of cell-free systems.

## Abbreviations

ATP: Adenosine triphosphate; 3-PGA: 3-phosphoglyceric acid; PEP: phosphoenolpyruvate; CP: creatine phosphate; Luc: *Firefly luciferase*; eGTP: enhanced green fluorescent protein; SDS PAGE: polyacrylamide gel electrophoresis.

## Competing interests

The authors declare that they have no competing interests.

## Authors' contributions

JS carried out all the experiments. VN carried out the background work of extract preparation and optimization. VN prepared the draft of the manuscript. All authors read and approved the final manuscript.

## Supplementary Material

Additional file 1 **Supplementary information for Shin and Noireaux "Efficient cell-free expression with the endogenous *E. Coli *RNA polymerase and sigma factor 70"**. supplementary information includes a list of the sequences, data on expression optimization (DTT, tRNA, amino acids, nucleotides), expression using pure *E. coli *RNAP for transcription, plasmid maps, expression of eGFP and eGFP-Del6-229 in *E. coli*. Click here for file
