# Supplementary material for: Efficient cell-free expression with the endogenous E. Coli RNA polymerase and sigma factor 70
Source: J Biol Eng. 2010 Jun 24;4:8. doi: 10.1186/1754-1611-4-8 (PMC3161345; doi:10.1186/1754-1611-4-8)
Supplement: Additional file 1 — Supplementary information for Shin and Noireaux "Efficient cell-free expression with the endogenous E. Coli RNA polymerase and sigma factor 70". supplementary information includes a list of the sequences, data on expression optimization (DTT, tRNA, amino acids, nucleotides), expression using pure E. coli RNAP for transcription, plasmid maps, expression of eGFP and eGFP-Del6-229 in E. coli. [file 1754-1611-4-8-S1.DOC]

**List of genes and regulatory parts used to construct plasmids**

Plasmid pBEST-Luc (Promega) was the original plasmid used in this work for cloning.

**PtacI:**

TTGACAATTAATCATCGGCTCGTATAATGTGTGGAATTGTGAGCGGATAACAATT

**OR2-OR1-Pr:**

TGAGCTAACACCGTGCGTGTTGACAATTTTACCTCTGGCGGTGATAATGGTTGCA

**UTR1:**

AATAATTTTGTTTAACTTTAAGAAGGAGATATA

**Luc:**

ATGGAAGACGCCAAAAACATAAAG……AAGGGCGGAAAGTCCAAATTGTAA

**eGFP:**

ATGGTGAGCAAGGGCGAGGAGCTGTTCACCGGGGTGGTGCCCATCCTGGTCGAGCTGGACGGC…………GTCCTGCTGGAGTTCGTGACCGCCGCCGGGATCACTCTCGGCATGGACGAGCTGTACAAGTAA

**eGFP-Del6:**

ATGGAGCTTTTCACTGGCGTTGTTCCCATCCTGGTCGAGCTGGACGGC……….GTCCTGCTGGAGTTCGTGACCGCCGCCGGGATCACTCTCGGCATGGACGAGCTGTACAAGTAA

**eGFP-Del6-229:**

ATGGAGCTTTTCACTGGCGTTGTTCCCATCCTGGTCGAGCTGGACGGC……….GTCCTGCTGGAGTTCGTGACCGCCGCCGGGATCTAA

**T500:**

CAAAGCCCGCCGAAAGGCGGGCTTTTCTGT

**Optimization of expression as a function of DTT**

|  |  |
| --- | --- |
|  |  |
| **Figure S1**  **Expression as a function of DTT concentration.** Top: maximum concentration of Luc synthesized as a function of DTT concentration with 0.5 nM plasmid pBEST-UTR1-Luc (left) and 5 nM plasmid pBEST-UTR1-Luc (right). White, gray and black for CP, PEP and 3-PGA buffers respectively (conditions: (CP) 3 mM of Mg-glutamate, 0 mM of K-glutamate, 1 mM of amino acids, 2% of PEG8000 for both concentration, (PEP) 2 mM of Mg-glutamate, 90 mM of K-glutamate, 1 mM of amino acids, 2% of PEG8000, (3-PGA) 1 mM of Mg-glutamate, 90 mM of K-glutamate, 1 mM of amino acids, 2% of PEG8000 for both concentrations). Bottom: kinetics of eGFP expression for no additional DTT (0 mM) with 0.5 nM plasmid pBEST-UTR1-eGFP (left) and 5 nM plasmid pBEST-UTR1-eGFP (right). Triangle, square and circle for CP, PEP, 3-PGA buffers respectively. In the case of eGFP, the optimal DTT concentration was 0 mM (conditions: (CP) 9 mM of Mg-glutamate, 0 mM of K-glutamate, 1 mM of amino acids, 2% of PEG8000 for both concentrations, (PEP & 3-PGA) 6 mM of Mg-glutamate, 60 mM of K-glutamate, 1 mM of amino acids, 2% of PEG8000 for both concentration). | |

**Optimization of expression as a function of tRNA**

|  |  |
| --- | --- |
|  |  |
|  |  |
|  |  |
| **Figure S2**  **Expression as a function of tRNA concentration.**  First row: maximum concentration of Luc synthesized as a function of tRNA concentration with 0.5 nM plasmid pBEST-UTR1-Luc at 1 mM of amino acids (left) and 1.5 mM of amino acids (right). White, gray and black for CP, PEP, 3-PGA buffers respectively (conditions: (CP) 3 mM of Mg-glutamate, 0 mM of K-glutamate, 2% of PEG8000, (PEP) 2 mM of Mg-glutamate, 90 mM of K-glutamate, 2% of PEG8000, (3-PGA) 1 mM of Mg-glutamate, 90 mM of K-glutamate, 2% of PEG8000). Second row: maximum concentration of Luc synthesized as a function of tRNA concentration with 5 nM plasmid pBEST-UTR1-Luc at 1 mM of amino acids (left) and 1.5 mM of amino acids (right). White, gray and black for CP, PEP, 3-PGA buffers respectively (conditions: (CP) 3 mM of Mg-glutamate, 0 mM of K-glutamate, 2% of PEG8000, (PEP) 2 mM of Mg-glutamate, 90 mM of K-glutamate, 2% of PEG8000, (3-PGA) 1 mM of Mg-glutamate, 90 mM of K-glutamate, 2% of PEG8000). The third row: kinetics of eGFP expression for no additional tRNA (0 µg/ml) with 0.5 nM plasmid pBEST-UTR1-eGFP at 1 mM of amino acids (left) and 1.5 mM of amino acids (right). Triangle, square and circle for CP, PEP, 3-PGA buffers respectively (conditions: (CP) 9 mM of Mg-glutamate, 0 mM of K-glutamate, 2% of PEG8000, (PEP & 3-PGA) 6 mM of Mg-glutamate, 60 mM of K-glutamate, 2% of PEG8000). The fourth row: kinetics of eGFP expression for no additional tRNA (0 µg/ml) with 5 nM plasmid pBEST-UTR1-eGFP at 1 mM of amino acids (left) and 1.5 mM of amino acids (right). Triangle, square and circle for CP, PEP, 3-PGA buffers respectively (conditions: (CP) 9 mM of Mg-glutamate, 0 mM of K-glutamate, 2% of PEG8000, (PEP & 3-PGA) 6 mM of Mg-glutamate, 60 mM of K-glutamate, 2% of PEG8000). | |

**Optimization of expression as a function of amino acids**

|  |  |
| --- | --- |
|  |  |
| **Figure S3**  **Expression as a function of amino acids concentration**  Top: maximum concentration of Luc synthesized as a function of amino acids concentration with 0.5 nM plasmid pBEST-UTR1-Luc (left) and 5 nM plasmid pBEST-UTR1-Luc (right). White, gray and black for CP, PEP and 3-PGA buffers respectively (conditions: (CP) 3 mM of Mg-glutamate, 0 mM of K-glutamate, 2% of PEG8000 for both concentrations, (PEP) 2 mM of Mg-glutamate, 90 mM of K-glutamate, 2% of PEG8000 for both concentrations, (3-PGA) 1 mM of Mg-glutamate, 90 mM of K-glutamate, 2% of PEG8000 for both concentrations). Bottom: kinetics of eGFP expression with 0.5 nM plasmid pBEST-UTR1-eGFP (left) and 5 nM plasmid pBEST-UTR1-eGFP (right). Triangle, square and circle for CP, PEP, 3-PGA buffers respectively. In the case of eGFP, the optimal amino acid concentration is 0.4 mM with 0.5 nM plasmid pBEST-UTR1-eGFP and 0.6 mM with 5 nM plasmid pBEST-UTR1-eGFP in any buffer (conditions: (CP) 9 mM of Mg-glutamate, 0 mM of K-glutamate, 2% of PEG8000 for both concentrations, (PEP & 3-PGA) 6 mM of Mg-glutamate, 60 mM of K-glutamate, 2% of PEG8000 for both concentrations). | |

**Effect of nucleotide concentration on cell-free gene expression**

Many different concentrations of NTPs have been used in the references used by Shin and Noireaux for their work.:

T7 transcription:

Sitaraman et al – 2004 [4]: 2mM for ATP and GTP, 0.85mM for UTP and CTP.

Kim and Swartz – 2001 [3]: 1.2mM for ATP, 0.85mM for GTP, UTP, CTP.

Kim et al – 2006 [22]: 1.2mM for ATP, 0.85mM for GTP, UTP, CTP.

E. coli transcription:

Zubay – 1973 [19]: 2.2mM for ATP, 0.55mM for GTP, UTP, CTP.

Three different sets of nucleotide concentrations were tested in the 3-PGA buffer.

Sitaraman et al – 2004 [4]: 2mM for ATP and GTP, 0.85mM for UTP and CTP.

Zubay – 1973 [19]: 2.2mM for ATP, 0.55mM for GTP, UTP, CTP.

Shin and Noireaux – 2010 [this work]: 1.5mM for ATP and GTP, 0.9mM for UTP and CTP.

In these three NTPs conditions, protein production is the same. Adjustment used in this study (1.5mM for ATP and GTP, 0.9mM for UTP and CTP) is slightly better than the two other systems.

**Figure S4**

Cell-free expression of eGFP with the 3-PGA buffer and three different nucleotides concentrations.

**Expression with pure *E. coli* RNAP**

Pure *E. coli* RNA polymerase saturated with sigma factor 70 was purchased from Epicentre Biotechnologies. Reaction was composed of:

- 50μl extract
- 30μl buffer 5X
- 6.25μl potassium glutamate at 3M (125mM final)
- 3.3μl Mg-glutamate at 500mM (11mM final)
- 17μl amino acids at 3mM (Roche mix), 0.33mM final each.
- 6μl pIVEX2.3d-Pr-eGFP at 50nM (2nM final)
- 22.45μl water

The reaction was split into 7*18μl samples, 2μl of E. coli RNAP were added. Reaction was carried out at 29°C on plate reader.

**Figure S5**

Cell-free expression of eGFP as a function of E. coli RNAP added to the reaction. No increase in protein production was observed.

**Plasmid maps**

Plasmid maps generated with ApE (A Plasmid Editor)

**Figure S6**

Plasmid map for pBEST-UTR1-Luc and pBEST-OR2-OR1-Pr-UTR1-eGFP-Del6-229-T500.

**Expression of eGFP and eGFP-Del6-229 in *E. coli*.**

**Figure S7**

Expression of eGFP (samples 1 and 3) and eGFP-Del6-229 (samples 2 and 4) with plasmid pBEST-UTR1 (PtacI promoter and UTR1) in *E. coli* strain JM109. Cells were grown at 30°C (samples 1 and 2) and at 37°C (samples 3 and 4). Cells were induced with IPTG for 2 hours before centrifugation. Cells were washed with PBS and the optical density was adjusted at the same value for all samples. Fluorescence intensity was measured on plate reader.
